# Ten simple rules for how you can help make your lab a better place as a graduate student or postdoc

**DOI:** 10.1371/journal.pcbi.1010673

**Published:** 2022-12-01

**Authors:** Matthias C. Rillig

**Affiliations:** 1 Freie Universität Berlin, Institut für Biologie, Berlin, Germany; 2 Berlin-Brandenburg Institute of Advanced Biodiversity Research (BBIB), Berlin, Germany

## Abstract

Lab teams are dynamic entities in which a lot depends on the principal investigator (PI) and the framework set by them. However, within these parameters, there is a lot of room for lab members to contribute to a happy and productive environment. Often doctoral students or postdocs (or other staff) may underestimate how much of a difference they can really make. Here are 10 simple rules on how to help make a lab a better place; these rules are mostly aimed at building better lab communities, where people help each other, look out for each other, and take an interest in critically questioning the status quo.

## Introduction

Principal investigators (PIs) and lab heads have the responsibility to ensure that their labs are healthy and productive places at which to work. Rules that help PIs foster healthy [1] and resilient [2] lab communities have been covered in previous work, and it is vital that adhering and valuing such aspects in lab culture becomes the norm. But PIs cannot achieve and maintain high-performing, collaborative research environments on their own [1]; they also need the engagement of people in the group, and it is a team effort to achieve a high level of productive collaboration and enjoyment when doing science [1,3,4]. This is what this paper is about: Even in a basically healthy lab environment, there is always room for improvement.

**Fig 1:**
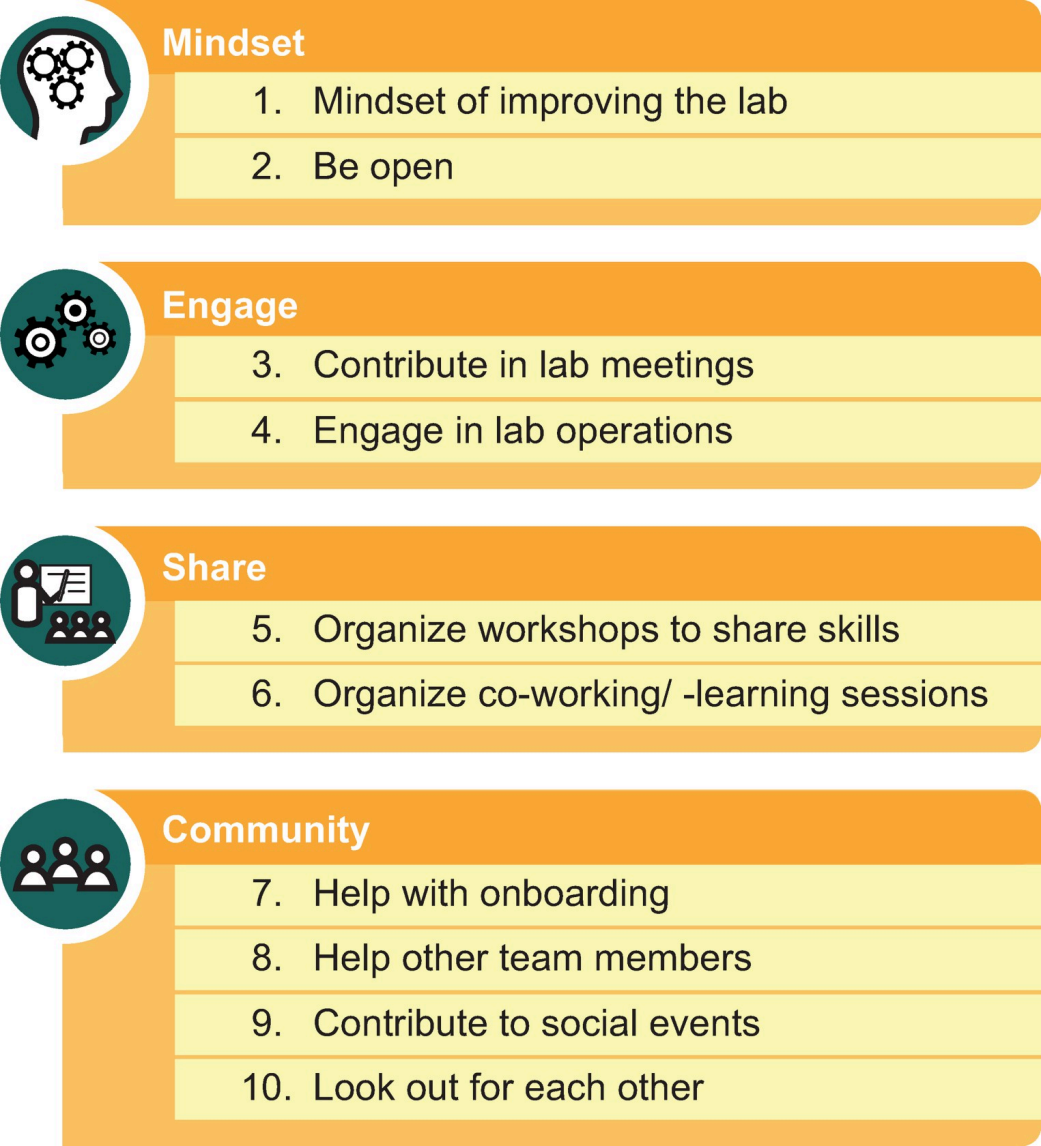
The 10 simple rules for how you can help make your lab a better place as a graduate student or postdoc, arranged by categories: “Mindset,” “Engage,” “Share,” and “Community”.

The 10 rules that follow (Fig 1), written by the PI of a lab after consultation with many team members past and present (as well as using feedback from presentations), are primarily aimed at graduate students, postdocs, lab managers, technicians, or undergrads, rather than PIs; but they should by no means be misunderstood as letting PIs off the hook when it comes to their duties. In fact, PIs should take note of these rules, create the necessary space for lab members and encourage the team to take ownership of that space. And of course, these rules can also not be a remedy for truly poor overall settings or horrible management.

It is important to note that many of the points that follow do not require a major effort on your part (the likely time commitment is noted after every rule). Also, following these “rules” does not just potentially improve the situation for other lab members, but also for yourself; in other words, they are not just altruistic measures, but also can specifically help your own progress in science (for each rule, these are listed under “Benefits”).

While most heads of labs will naturally welcome suggestions on how to make a lab work better, it is still essential to be in close dialogue [4]: Of course, the PI needs to agree with the various changes if they affect the way the lab is operating. This dialogue is important, because not all PIs will likely be as enthusiastic when it comes to this topic, so it is also important to proceed with caution. Securing this buy-in from the PI is thus an essential prerequisite for following the “rules” that follow, in particular for actions that influence the lab’s culture and operations. Otherwise, this may fail and potentially lead to lab members feeling like they have failed in their role.

## Rule 1: Adopt a mindset of improving the lab

In essence, this means following a “growth mindset” (as opposed to a “fixed” mindset; see [5] for a discussion) for the lab group as a whole. It is as simple as not taking for granted how things are currently, and asking how they can be better. Improvements do not need to be drastic changes, sometimes it is little things that can be improved, and many of such small steps can make a big overall difference with time. Perhaps, it is about how the lab journal club is organized, or about how lab procedures can be adapted, how lab logistics can be better orchestrated, how group discussions can be more meaningful, or about team activities. Maybe there can be a discussion leading to the drafting of a “lab philosophy” document, jointly authored by the PI and lab members, or similar discussions about best publishing practices and how to engage more deeply in open science. Lab members could also initiate or facilitate discussions about diversity, equity and inclusivity (DEI), and become involved in drafting lab policy documents on these issues. Another idea is to encourage the PI to carry out an anonymous lab survey to gauge how the lab is operating [6].

In my group, for example, there was a team member-driven activity (meaning this was not my idea), where a smaller group of people met without me as the PI, to discuss suggestions for improvement. The group met regularly for a while (weekly or monthly) and then reported back to me, having discussed more general topics and specific suggestions. For example, they discussed in general terms how lab meetings could be run more effectively, with people contributing more. One specific suggestion was to offer time slots not just for 45-min presentations, but also short meeting slots (5 to 10 min), for which people could sign up as well. How this is organized will depend on your lab, but make sure it is in consultation with your supervisor. This latter point is crucial, since not everything in the lab will be up for change, and this can only be done within the framework set by the PI. Before embarking on this exercise, it may therefore be useful to have an open discussion with the head of the lab about what parameters are fixed.

Time investment: low to medium. Benefit: Feeling more “ownership” about how the lab is run.

## Rule 2: Be open

Openness can be about anything, about new methods that the lab has not established yet, about interactions with other groups or disciplines, about ideas and thoughts, about being open with data and lab resources, or new tech tools [7]. If you are open to new things, new opportunities might also open up to you, and you contribute actively to a very positive aspect of lab culture that cannot be prescribed.

As an example, I always liked this story of an artist we hosted in our lab: “I came in the door in the morning, and I didn’t even make it to my desk until in the afternoon, because people wanted to show me their experiments and what they were working on.” It is one thing to have an artist-in-residence in the lab [8], but it becomes transformative if team members are open to the new experience and embrace it.

Time investment: low to medium. Benefit: New insights and opportunities.

## Rule 3: Engage and contribute in lab meetings

Lab meetings can be the best of times, but can also be a drag, namely when people do not contribute, or when it is always the same few teammates speaking up. Journal clubs or other lab meetings are really for everybody’s benefit [9]: all ideally learn from them by discussing the topic at hand. Such meetings, especially in large groups, can be intimidating. But it is really worth it to engage in the discussion and contribute with insights or even with questions. A broader contribution makes these meetings more fun, productive, and insightful for all. So come prepared (e.g., having read the paper for journal club) and offer your perspective. If you have a question, for sure a bunch of other people also have the same question. If your lab does not have lab meetings yet, perhaps you can initiate them, also jointly with other like-minded groups: their value is immense.

Time investment: low. Benefit: Discussions in lab meetings frequently lead to new ideas and collaboration.

## Rule 4: Be engaged in the operational side of the lab

Labs only work because some people take care of the ordering, the administration, the organization of work stations, the cleaning, and other tasks. Of course, it is always expected that you do your part, but you could also take more of an interest in how things work behind the curtains, and thus help generate ideas about how they could become better (see Rule 1). Maybe you could look into how to make tasks less energy consuming, more environmentally friendly by using substitute chemicals, or how to administratively organize things better. You can only do that if you take an interest in how things work in the first place.

Time investment: low. Benefit: The insights you gain can be useful in your next position or when you set up your own lab.

## Rule 5: Spread skills by organizing small sessions or workshops

Perhaps you have mastered a new statistical package, or a new lab skill, or you have tried a time management app, and what better way to use that knowledge than to spread it to your teammates. This can take the shape of a short lab meeting session where you present your new insights or also a workshop for part of a day. This promotes an atmosphere of knowledge sharing, from which you can also profit when others do the same. While this does take time to prepare, you also build your presentation skills, organize better what you know, and you may be winning collaborators for your work, for example, when you share that you know a certain analytical method. For example, in our group, 1 lab member gave a short session on a statistical technique, passing on knowledge gained from an external training workshop to the lab group; this signaled to the lab that this knowledge is now in the group, led to much feedback to the person presenting, and the person also felt that having presented and organized the contents had bolstered his own command of the subject matter. Another example was about time management, a topic on which we had several presentations by different people. This led to interesting follow-up discussions and also to lab members taking up certain routines or using certain methods or apps.

Time investment: medium. Benefit: Fortifying your skills, winning collaborators.

## Rule 6: Organize co-learning/co-working sessions

While Rule 5 is about 1 person sharing their knowledge, this rule is about learning together. Often, new challenges come up in scientific work, such as new methods to learn or new concepts to tackle. Science need not be a lonely endeavor, but often progress can be faster in small teams where people work towards a common, specific goal (such as “let’s learn how to use this statistical package” or “let’s implement this new lab method”). Peer mentoring [10] can be an effective teaching tool and can supplement the more traditional one-on-one, hierarchical model.

Another way to work together with a very low threshold is to organize co-working sessions, such as for writing. The sense of accountability simply provided by the presence of others (online or in person) can be a driver of progress, and the community created can also provide comfort when things do not work out as well. For this, all you need is to set a time to meet.

Time investment: low to medium. Benefit: Achieving faster progress in difficult questions or tasks.

## Rule 7: Help out with onboarding

Arriving in a new lab and environment (or even country) is a challenging time for almost anybody, with lots of things to think about [11]. Your institution will likely provide some help with orientation, and your PI will likely introduce the new person and show them around. However, it makes all the difference when there is an active offer of help from team members to share with the newbie the best places to eat or to help with getting settled in the new lab. This can entail small gestures, but also be more involved, such as teaching the new person some lab methods or introducing them to the lab culture [11]. At the very least, you will earn the gratitude of someone who has just arrived.

Time investment: low. Benefit: Winning a collaborator and/or a friend.

## Rule 8: Help out other team members with their work

Often research projects include crunch times when things are hard to manage by any one person alone, such as setup of experiments with a large number of experimental units, harvests, time-critical lab measurement campaigns, or also other tasks, such as screening a large body of literature for a systematic mapping exercise. In such situations, it is really excellent to offer help, even for just a few hours, as it releases stress from the person running the study. Offering help can establish a reputation of generosity, and when it is your time to tackle a critical task, people will likely want to repay the favor. This way everybody wins.

Time investment: low to medium. Benefit: Getting help when it is your turn; establishing a positive reputation.

## Rule 9: Contribute to social events

Lab outings, provided they are planned as inclusive events (for example, to also allow parents or caregivers to attend, in settings that are comfortable for everyone), are the times when you can talk with your teammates and experience them in a non-work context. Perhaps you like the same books, sports team, or movies, and the relaxed atmosphere can also help make research connections, especially in larger teams. It is making such connections that lead to a lab being more than the sum of its parts, making them more collaborative and hence, overall better places. So, do not miss out on these. Such events can help build community and establish lasting friendships. Maybe you also want to take a more active role and bring some food to share or your guitar, or make suggestions for places to visit.

Time investment: low. Benefit: Making connections.

## Rule 10: Look out for each other

Graduate school and also postdocs can be stressful times and mental health problems are not uncommon [12,13]. So, even if we do not usually think about this, chances are one of your teammates will run into problems as well, and you, as a peer, are very likely to discover it before your PI, especially if you watch out for signs of changes in behavior. In fact, it is often peers that provide mental health support [14]. You are not a psychologist of course, but just having someone to talk to, and showing that you care can be extremely helpful. And perhaps, you can help them also by pointing to resources that can offer professional assistance. Helping others can be a stressful experience itself, and thus potentially negatively affect the helper [14], and so it is important that you see your role primarily as having an open ear, and that you refer your lab mate to professional help, if needed.

Time investment: low. Benefit: Gratitude of those who you helped.

## Conclusions

Lab teams are dynamic entities that work best and most sustainably when there is a good community spirit. This sense of community often leads to better performance, and this in turn will feed back to a better sense of community (“we achieved this”). “Better labs” includes many aspects in this context, but almost all the 10 Rules are aimed at forming a better community, where people help each other, look out for each other, and interact productively. This can, for example, lead to more joint experiments within the lab, incorporating different skill sets, more joint publications among team members, the generation of more and better ideas, and greater team resilience when faced with negative outside events.

Certainly, a lot will depend on the PI, and this is also why PIs should take note of the Rules offered here, but within this framework team member contributions can make a huge difference. Doctoral students, undergraduate students, technical staff, or postdocs perhaps often underestimate how powerful their contributions to lab culture can be. Such contributions do cost time, but typically not that much, and nobody will contribute in all possible ways outlined above, simply because people have different interests and skills. What you reap in return is a better lab, and each of the Rules also comes with specific benefits also for yourself, not just for the group.
